# Economic sanctions and academia: Overlooked impact and long-term consequences

**DOI:** 10.1371/journal.pone.0222669

**Published:** 2019-10-01

**Authors:** Louise Bezuidenhout, Ola Karrar, Javier Lezaun, Andy Nobes

**Affiliations:** 1 Institute for Science, Innovation and Society, University of Oxford, Oxford, England, United Kingdom; 2 Department of Statistics, University of Khartoum, Khartoum, Sudan; 3 INASP, Oxford, England, United Kingdom; East Carolina University Brody School of Medicine, UNITED STATES

## Abstract

Financial sanctions are often thought of as the “soft alternative” to armed conflict and are widely used in the 21^st^ century. Nonetheless, sanctions are often criticized for being non-specific in their action, and having impact beyond their intended remit. One often-overlooked area affected by sanctions are academic systems of research and education. Sanctions place “invisible barriers” for research in these countries by limiting access to necessary resources and curtailing their effective use. In this paper we present a national survey of Sudanese academics focused on the impact of 20 years of economic sanctions on their work. It identifies key areas of academic research and education that have been impacted by international sanctions. Moreover, these data highlight how the impact of sanctions on academia is likely to persist long after they are formally lifted. The paper concludes by problematising the current interpretation of *jus post bellum*, or moral behaviour after conflict. It suggests that the responsibility to make reparations in the form of support for academic systems applies to countries who impose economic sanctions.

## Introduction

This study examines the impact of financial sanctions on higher education and academic research. The introduction briefly introduces the concept of sanctions to the reader, and outlines why they are an object of concern for robust academic infrastructures.

### Sanctions as an alternative to war

Since World War I, sanctions have become increasingly viewed as a liberal alternative to war [1]. Modern sanctions can be largely grouped into two different areas: weapons-trade restrictions and economic restrictions. Economic sanctions, the focus of this paper, can be implemented with varying intensity and scope. For example, the entire opposing economy may be targeted or just one critical sector [1]. Regardless of the type and style of implementation, economic sanctions may be best understood as actions aiming to lower the aggregate economic welfare of a target state through a reduction in international trade.

Since the end of the Cold War the use of sanctions has risen considerably, and the tendency has become even more marked since the turn of the century. For instance, as quoted in Drezner, “between February 2014 and February 2015, the US introduced or amended twenty different sanctions programmes” [2]. While there have recently been fewer comprehensive, multilateral sanctions introduced, states still engage in unilateral or bilateral broad sanctions and multilateral, “targeted” sanctions [3]. The latter could include asset freezes, travel bans and smart trade sanctions.

The US Office of Foreign Asset Control (OFAC), for example, interprets comprehensive sanctions as prohibiting all transactions between the sanctioned country and the US (except in special circumstances where a license is granted by OFAC). This means that there can be no imports, exports, financing of goods, distribution of technology and services, or trade brokering between American citizens and the sanctioned country [4]. Countries currently under comprehensive, multilateral sanctions include the Democratic People's Republic of Korea, Iran, Cuba, and Syria. Until recently, this list also included Sudan. The US also maintains other, more “targeted” (i.e. counter-terrorism, counter-narcotics) sanctions against specific individuals and entities. These programs may encompass broad prohibitions at the country level as well as targeted sanctions [5].

### Sanctions as indiscriminate tools

Despite their widespread use, sanctions are often criticized for failing to realize their policy goals [1], [3]. In a review of sanctions literature, Mack and Khan write that “the only real disagreement in the contemporary sanctions literature relates to the degree to which sanctions fail as an instrument for coercing changes in the behaviour of targeted states. No study argues that sanctions are in general an effective means of coercion, although individual sanction regimes can and sometimes do succeed” [6]. They rarely lead to regime changes or reform [7]. The sanctions regime used against Saddam Hussein’s Iraq, in particular, have been subject to widespread criticism. It has been noted that “nearly everything for Iraq's entire infrastructure—electricity, roads, telephones, water treatment—as well as much of the equipment and supplies related to food and medicine has been subject to Security Council review” [8]. Indeed, Gordon highlights how the US definitions of “dual use” and “weapons of mass destruction” led to the argument that everything, from water pipes to laundry detergent or children vaccines, could be used to produce weapons. This understanding enabled the US to prevented critical humanitarian goods from entering Iraq [9].

The potential of sanctions to trigger broad humanitarian crises relates not only to an expansive interpretation of security, but also to the generally indiscriminate nature of economic sanctions. The difficulty of limiting economic impact to a single locus means that the effect of sanctions can spread far beyond their intended reach, leading to widespread suffering [3], [10]. It has been observed that sanctions often lead to an increase in inequality in the targeted county, often harming rural and non-industrialized areas disproportionally, and that they often thwart humanitarian and aid efforts unrelated to the targeted state’s political behavior. Moreover, the effects of sanctions persist after they are lifted, as they can have a negative impact on the cost of reconstruction and development.

### Sanctions and academia: Why the concern?

The indiscriminate effects of sanctions on national infrastructures has been noted in many situations. However, as the world becomes increasingly interconnected and interdependent, it is easy to see how economic sanctions can have widespread impact on areas not traditionally associated with economic activity. In this paper we consider the impact of economic sanctions on one such area—academic research and education.

Modern academic research and education is reliant on an international network of collaborators, online information, equipment and reagent suppliers, and international travel. Moreover, as national actors increasingly promote research, development and innovation (RDI), the boundaries between research and commercial ventures, public, private, and state activities, or education and innovation (in terms of activities, equipment utilized and research outputs), are increasingly blurred. This blurring has been epitomized by the “triple helix” model of university-industry-government relations proposed by Etzkowitz and Leydesdorff [11]. Governments, they suggest, “are offering incentives, on the one hand, and pressing academic institutions, on the other, to go beyond performing the traditional functions of cultural memory, education and research, and make a more direct contribution to "wealth creation. Moreover, multi-national institutions such as the European Union, the World Bank and the U.N. are also “moving to embrace concepts of knowledge based economic development that bring the knowledge, productive and regulatory spheres of society into new configurations” [11].

The embeddedness of modern academia within the national and international economic exchange necessarily raises concerns about the imposition of sanctions. Such concerns must extend beyond recognizing the restrictions on importing equipment or finances from abroad. Indeed, modern sanctions do not only control physical trade, but also online activity. Thus, the same sanctions that stop academics from purchasing laboratory equipment may also be responsible for limiting their access to online data, if the company hosting that data is based in a country that is imposing sanctions. Similarly, some academic publishers in the US are known to have refused submissions from Iranian and Sudanese authors due to by threats of fines of up to $1 million and imprisonment of up to 10 years by the US treasury. Even after legal action caused a revision of these rules, the vagueness of the existing policies have still led to some reluctance on behalf of publishers to accept papers from authors in countries under sanctions [12]. In this way, the reach of sanctions insidiously extends beyond the intended target to affect all areas of academia.

By introducing barriers in many areas of academic activity, sanctions can be seen to influence what research is done, how it is disseminated, and how students are educated. As many of the countries under long-term sanctions have relatively small and fragile academic communities and low rates of investment in STEM, the impact of sanctions can be devastating. One of the major difficulties in raising these concerns is the lack of evidence from academic communities under sanctions about the impact of these restrictions on their academic activities.

Within the literature on sanctions, there are only a handful of references to the impact of sanctions on academic systems. For example, the aforementioned sanctions on Iraq which had impacted on all areas of Iraqi life and infrastructure, also profoundly affected education at primary, secondary and higher levels, which had previously seen unprecedented improvement before the sanctions [13]. This has inevitably had a knock-on effect on research capacity and post-conflict research and reconstruction, for example childhood trauma research [14].

In Iran, US sanctions are known to have harmed scholarly and scientific activities on several levels: restricting international collaborations; travel opportunities for conferences and workshops; and international collaborations, inevitably reducing scientific output [15]. Some researchers have reported problems publishing in international journals for political, not scientific reasons [16], [17], in some cases because of confusion over the authors’ employment by the government [18]. Scientists have also experienced problems with paying for society subscription and event registration [16]. Whilst there is some evidence of growth in scientific output since sanctions were relaxed, this may be partly down to the investment and growth into Iranian-published scientific journals [19].

Serbian and South African academics who worked during periods of sanctions (1992–2001 and 1960–1990 respectively) report not only severe restrictions on travel, international collaboration, access to resources and inability to publish, but also notable psychological effects of isolation [20–23]. Post-sanction recovery in these countries didn’t occur immediately, but was driven by transition into a free market global economy. In the case of Serbia, external support was particularly vital as academics had largely missed out on the digital information revolution [20], [24], as well as suffering the effects of hyperinflation and brain drain [21]. Similar to Iran, research output has been partially improved by the growth in national publishing outlets and government incentivising of publication outputs [21].

The literature on the long-term impact of sanctions on the growth trajectory of academia after sanctions are lifted is thus very limited and requires further investigation. The coming section empirically addresses these issues, using a survey conducted amongst Sudanese academia as a case study.

## Study objectives

While there has been anecdotal discussion about the impact of sanctions on Sudanese academia, there is a paucity of empirical evidence on the subject. This study aimed rectify this oversight by surveying Sudanese academia regarding their perceptions of the impact of sanctions on their work. It is important to highlight that while the survey responses are are self-reported perceptions of impact, they nonetheless provide insight into the impact of sanctions on daily research and educational activities.

## Methodology

### Selection of case study

Sudan is one of the few countries that have been subjected to a long-running system of comprehensive sanctions. In 1993 Sudan was designated as a state sponsor of terrorism, leading to a suspension of United States (US) Embassy operations in 1996. In 1997 the US imposed comprehensive economic, trade, and financial sanctions against the country [25]. The sanctions included a fairly comprehensive trade embargo, a freeze on government assets, and tight restrictions on financial institutions dealing with Sudan. The sanctions prohibited any transactions using US currency or products, and stopped any business which operated in the US from trading with Sudan. This ban covered everything from airplanes to vital health equipment. Similar measures were subsequently introduced by, amongst others, the European Union and the United Kingdom. Moreover, in 2005 the United Nations Security Council passed Resolution 1591 in response to the conflict in Darfur, imposing an embargo on the sale or supply of arms ‘and related material,’ as well as a travel ban and asset freeze on several individuals.

Studies on Sudan show evidence of widespread impact of sanctions on areas integral to national development and economic recovery. These include social entrepreneurship initiatives, domestic education, medical industry, civil infrastructure, personal and family finances and local businesses [25]. Moreover, there is increasing evidence that sanctions have also had an impact on the international recognition of Sudanese certifications. Malik notes, for instance, that “required [computing] certifications necessary to advance careers and establish legitimacy are not available in Sudan—Google does not allow their certifications to be received in the country. As a result, researchers and students are forced to either work uncertified, or travel outside of the country to receive the certification” [25], [26].

In 2017 the sanctions against Sudan were partially lifted by the US. Nonetheless, it is likely that Sudanese academia will experience the same long-term impact of the international sanctions as discussed above. The lack of a comprehensive understanding of the impact of over 20 years of sanctions on the academic regime also makes it difficult to highlight key areas requiring targeted intervention, funding and capacity building.

### Limitations of study

This study was carried out in 2018 while President Bashir was still in power, but after the US started to lift the sanctions in 2017. The political situation in Sudan has changed dramatically since this study was conducted, more of which will be discussed below.

The effects of political instability and sanctions are, of course, very difficult to disentangle, and a significant overlap in both action and effect is to be expected. However, sanctions can be seen as uniquely contributing to academic *interactions beyond borders*. It is this area that the rest of the paper will focus on, namely how sanctions have affected and continue to affect the ability of Sudanese academics to engage with the global science system.

### Methodology

#### Ethics and piloting

In order to gather data around this topic, we designed and disseminated a survey with questions relating to daily research practices, access to online resources and engagement with online resources. The survey was originally developed in English and translated into Arabic. Ethical approval for the dissemination of the survey was granted by the School of Anthropology and Museum Ethnography Research Ethics Committee at the University of Oxford (Ref No.: SAME_C1A_17_103).

The survey was hosted on SurveyMonkey and distributed electronically to teaching staff via institutional emails. A link for the questionnaire was also shared through a number of scientific and academic Sudanese networks in social media websites, mainly Facebook and Twitter, recommended by number of active researchers to involve young researchers who are actively interacting through these platforms. The purpose of the survey was clearly outlined on the survey landing page, together with details of data protection and re-use. Potential participants were informed that participation in the survey was entirely voluntary and anonymous. This introductory page also stated that participation in the survey was taken as consent for the use of the data as outlined.

The survey was piloted at the University of Khartoum with 15 academics representing a diversity of gender, age and discipline. Comments from the pilot were incorporated into the final survey design.

#### Inclusion criteria and dissemination

To determine a roll-out strategy, consultations were held with the director of research in the Ministry of Higher Education and Scientific Research (HESR) and his deputy, and with a group of senior professors in different research domains. According to their advice, we targeted the three top-ranked universities in Sudan as well as research institutes with active research groups and well reputed research leadership and networking. These universities and institutes contain the majorities of senior professors contributing to scientific publication and training of younger generation of master and PhD students.

The survey had very broad inclusion criteria, and was open to any individual who was employed as full/part-time academic staff (postdoctoral researchers, lecturer or higher academic grade) at a Sudanese academic institution. It was also open to full/part-time postgraduate students (Masters or PhD at Sudanese universities) and individuals working in governmental/commercial institutions who are involved in research activity in Sudan. Exclusion criteria were individuals not currently engaged in research activities, not currently employed in Sudan, or engaged only in undergraduate studies. Demographic data was collected about age, gender, discipline, years of research activity and research institution so as to allow selected analyses.

The voluntary survey was live for a three-month period between November 2017 and February 2018. The survey had broad inclusion criteria, requiring participants to be employed by an academic institute, resident in Sudan and in possession of at least an undergraduate degree. In total, 328 completed responses were collected. The major research groups represented were in the fields of health sciences, life sciences, natural sciences, social science, engineering and business. The demographics of the study population are presented schematically below.

#### Data management

All data from the survey was fully anonymised at point of collection. The data was stored securely on a password-protected cloud storage facility and backed up on the password protected University of Oxford server. Only the authors of the study had access to the raw data. In keeping with the agreed project outline, a summary of the survey data has been archived at the Open Science Foundation (https://osf.io/9tw7x/), and the raw data will be destroyed 2 years after the completion of the project publications.

## Results and discussion

### Demographics

**Fig 1:**
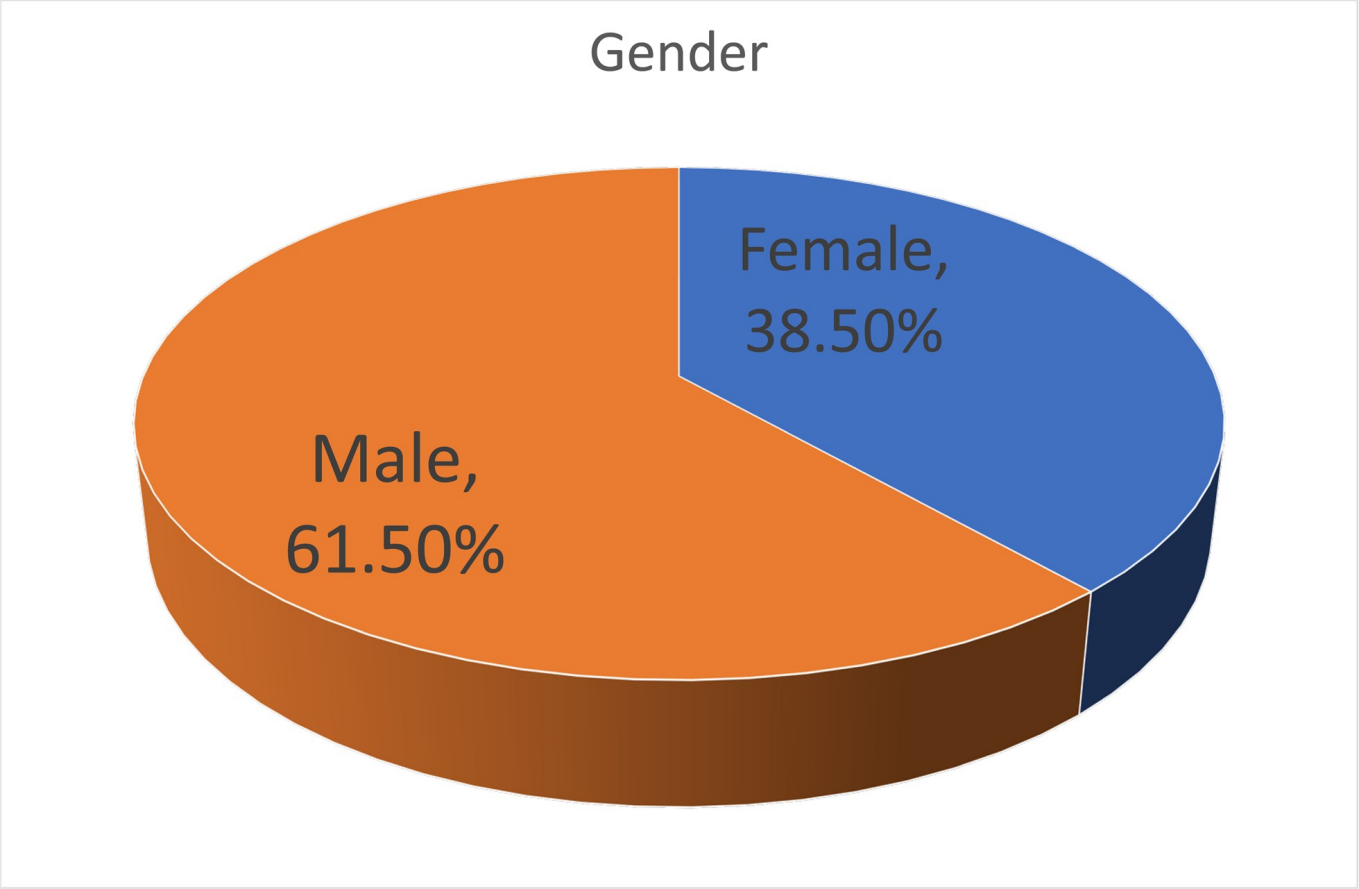
Gender of respondents.

**Fig 2:**
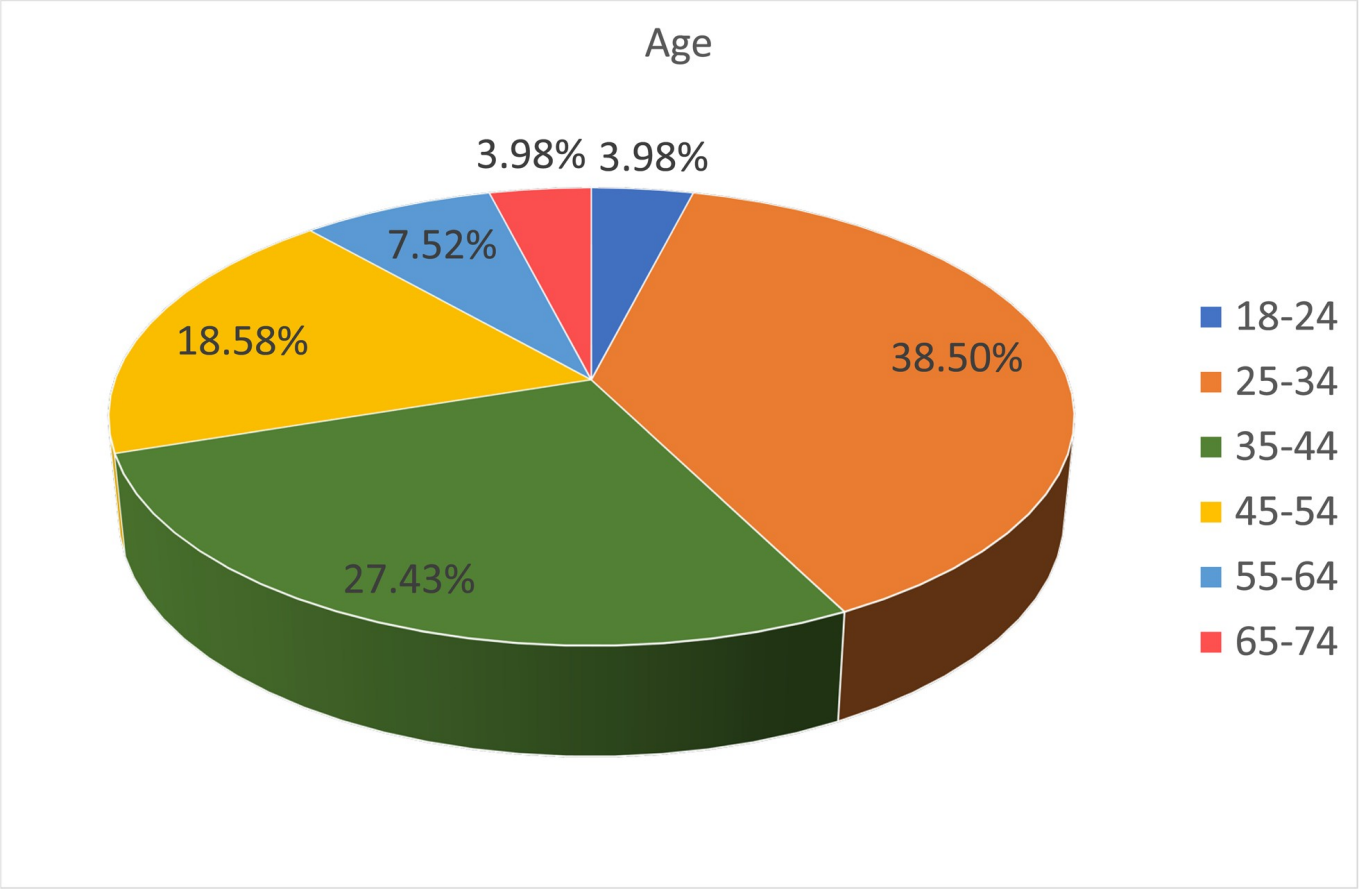
Age of respondents.

**Fig 3:**
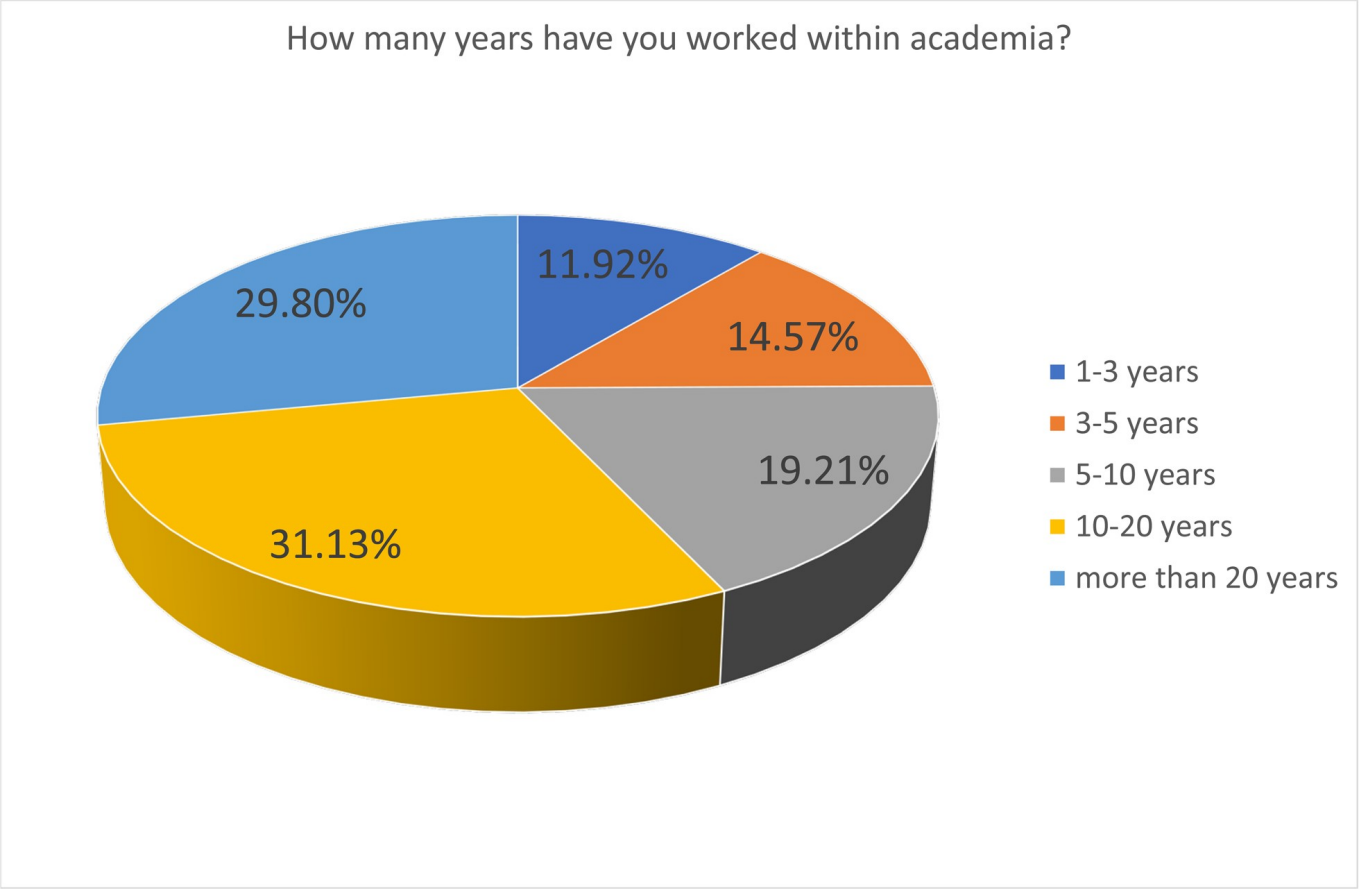
Years of experience in academia.

**Fig 4:**
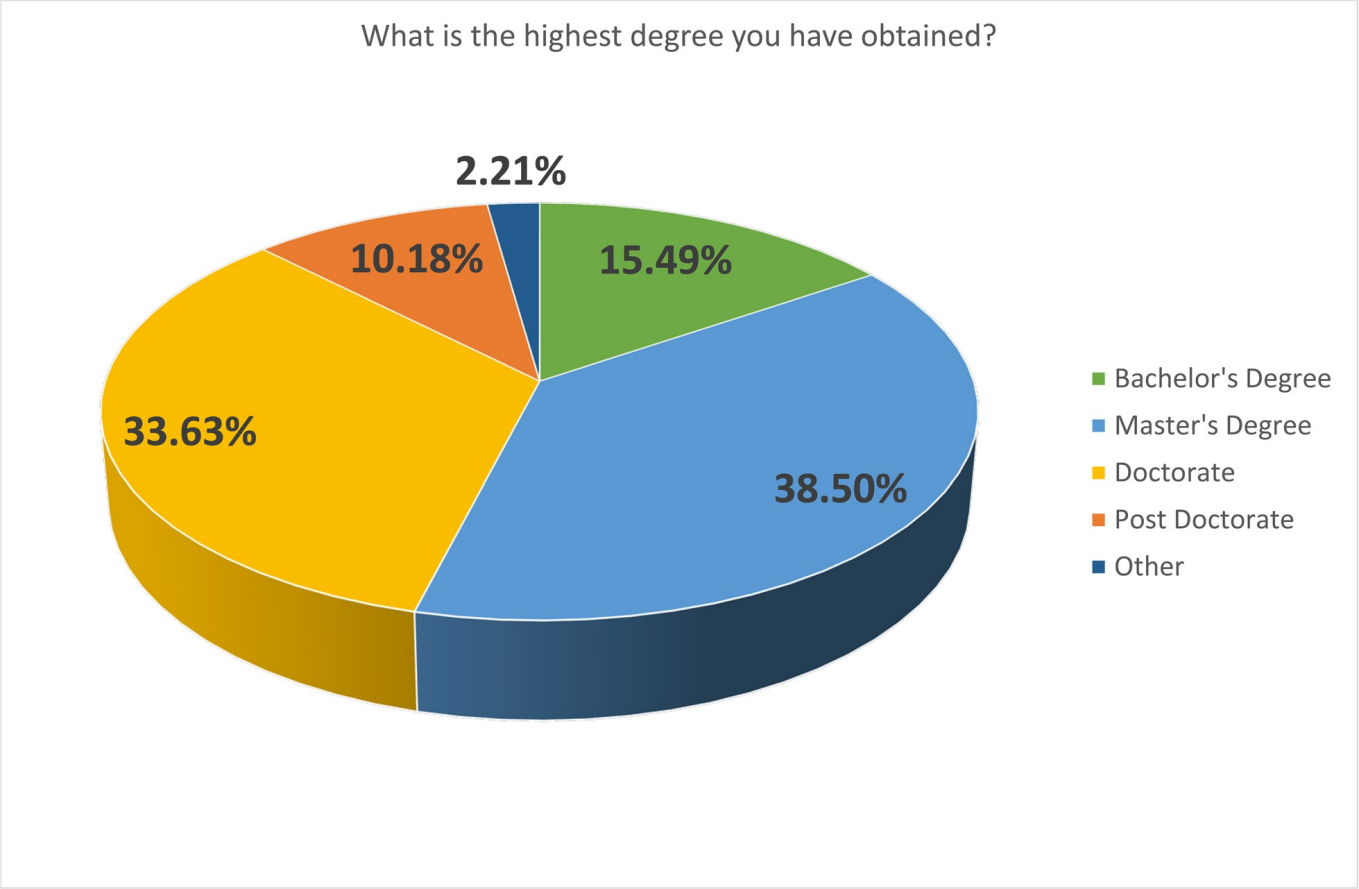
Highest degree obtained.

The respondents were predominantly male (61.5%) (Fig 1), and relatively young, with 42% under 35 age bracket, a further 27% in the 35–44 age bracket and the remaining 31% were over 45 (Fig 2). In terms of experience, 26% had spent less than 5 years in academia, 19% between 5 and 10 years, and the remaining 54% had over 10 years of experience (Fig 3). Those with a doctorate made up 43% of participants, with 38% having achieved a Master’s and 15% a Bachelor’s degree (Fig 4).

The survey questions were grouped into two main sections. The first aimed to examine the perceived impact of 20 years of sanctions on all areas of academic activity. The second section asked respondents to identify areas that needed further investment in order to facilitate the effective growth of Sudanese academia in the post-sanction era.

**Fig 5:**
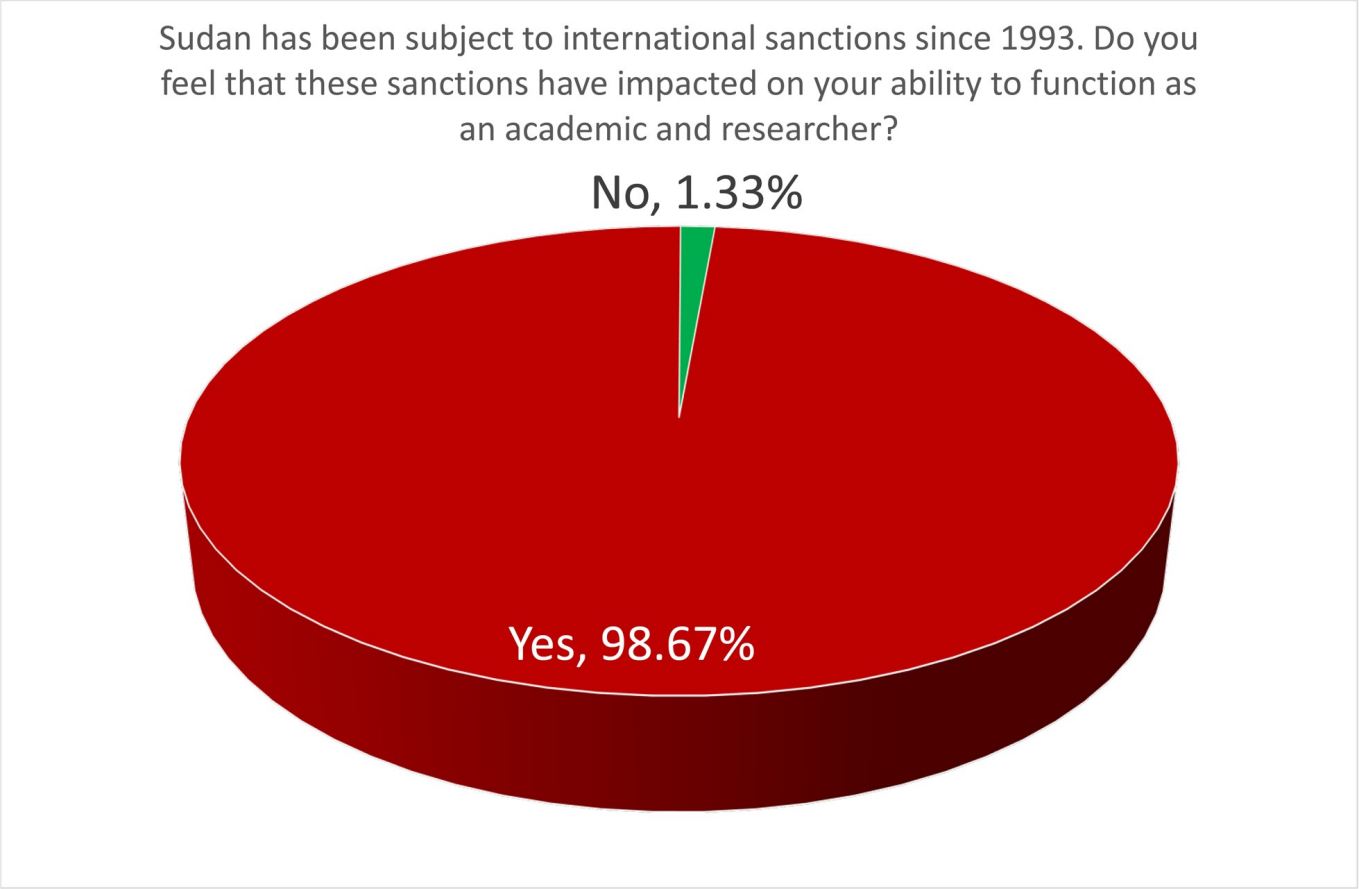
Did sanctions impact on respondents ability to function as an academic and researcher?

One of the first questions on the survey was whether respondents felt that sanctions had impacted their ability to function as an academic and researcher. As Fig 5 demonstrates, a compelling 98.67% of respondents answered “yes,” unequivocally supporting our assumption that the impact of sanctions was significant and worthy of detailed study.

### The impact of sanctions on Sudanese academia

#### Academia and the economic climate under sanctions

When the survey was designed, it was recognized that separating out the impact of sanctions and the general economic climate would be very difficult. Indeed, as the sanctions were intended to cause economic downturn, sometimes these “cause and effect” assignations were one and the same. Indeed, it is highly likely that the imposition of sanctions and the resultant economic downturn led to curtailed budgets for academia and less investment in academic infrastructures. In consequence, some of our results will be similar to reports from other low/middle-income countries that are not necessarily under sanction. The issue of how limited financial investment in academia leads to lack of equipment, reagents and research infrastructures across LMICs has been researched and discussed widely (10,11).

Readers might therefore be tempted to dismiss the reported concerns about economics as “general problems for LMICs”. Nonetheless, countries subjected to extensive sanctions differ from other LMIC economies in several respects:

- Inability to access currency: banking restrictions curtail purchasing with foreign currency as well as access to funds held abroad;
- Lack of equipment and reagents: funds are not necessarily absent, but are difficult to spend
- Purchasing power: sanctioned economies such as Sudan may experience hyper-inflation that can lead to currency devaluation and lower purchasing power
- Addressing inequalities: researchers may not be eligible for funds or support to address resource shortages

These distinctions were evident in the responses for the question: “select the top three challenges you experience while managing a research grant under sanctions”. The most frequent response (65.22%) was “banking restrictions complicate research-related purposes”. The next two responses were “distributing the grant funds to cover necessary core purchases such as hardware and software” (45.7%) and “accessing funds from funding bodies” (39.1%). These responses clearly show that researchers operating in sanctioned economies face difficulties in mobilizing and utilizing available funds.

#### Funding under sanctions

Only 1/3 of respondents said that they had received research funds in the period between 1993 and 2017. The majority of respondents who said that they had received funding were recipients of national funding under $10000. This would suggest that most of the research projects would be short/medium length and collect smaller datasets. This has widespread complications that are often under-recognized that shape research practices. Necessitating academics to commit to short, relatively small research projects often causes difficulties for the individual researcher in forging a coherent body of research and data. This can cause them to be excluded from future collaborations and from contributing high-impact publications to their field (12).

It is also likely that lack of access to international funds and funded research networks has long-term impacts on the diversity of the research landscape. This survey data highlights the need for a comprehensive review of Sudanese publications and funding in the sanctioned period. This will enable the government and international funding community to identify areas that are critically under-funded and under-represented within the Sudanese academic landscape.

#### Access to equipment, hardware/software under sanctions

Academic research and education are increasingly reliant on hard/software and other equipment, much of which is made in the Global North. It can become very difficult to procure the necessary equipment from countries under sanction, meaning that the sanctions can have significant impact on the style, content and continuation of academic research and education. Moreover, as the rate of technological innovations speeds up, sustained periods of sanctions can leave academic communities considerably out of date.

**Fig 6:**
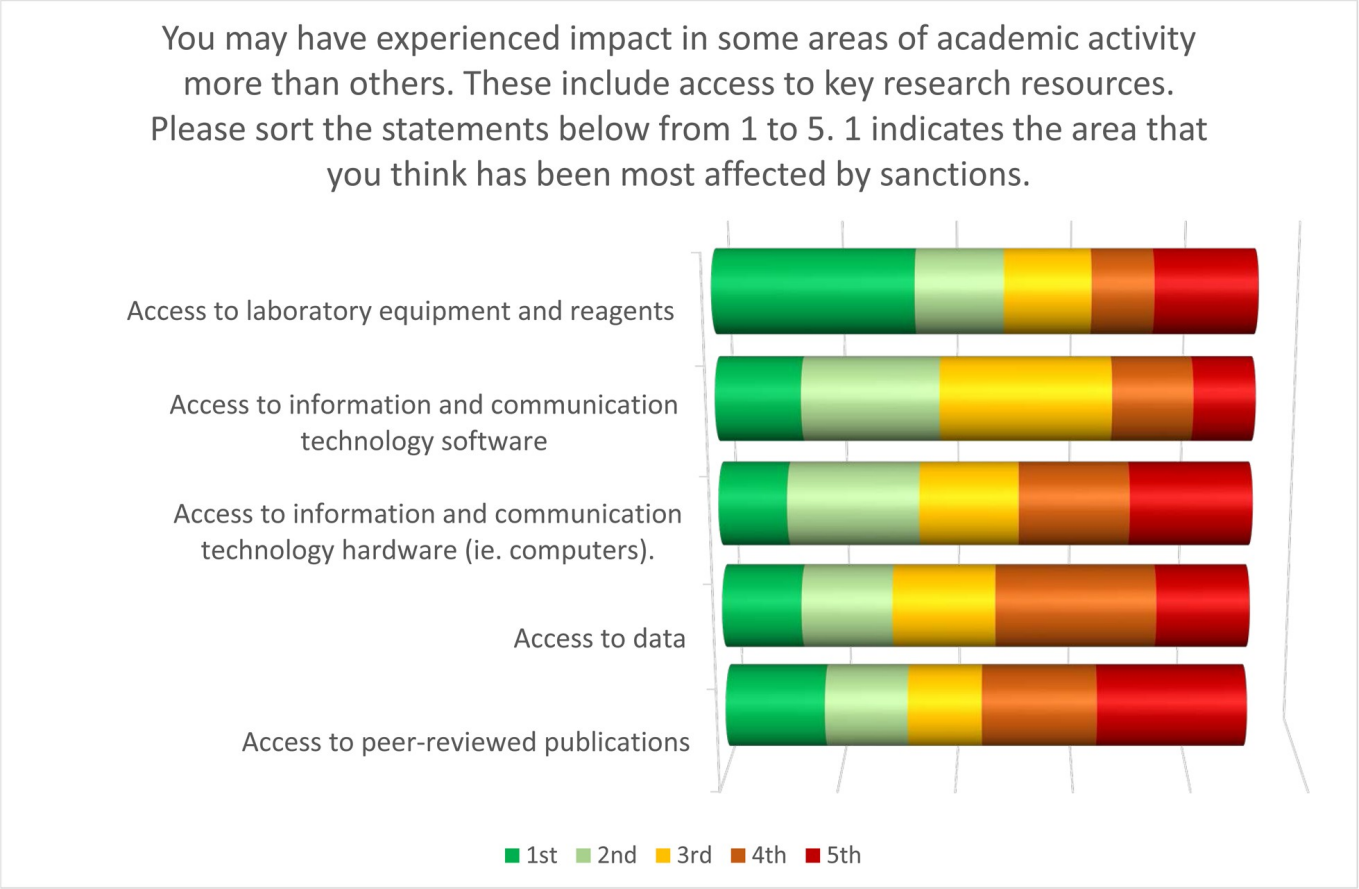
What areas of academic activity have been most impacted by international sanctions?

When asked respondents to rank the areas in which sanctions had most affected their academic activities, the responses showed considerable diversity (Fig 6). This suggested that the sanctions had wide-reaching and pervasive influence. Of all options, however, “access to equipment and reagents” was ranked the most impacted, while “access to peer-reviewed publications” was the lowest.

**Fig 7:**
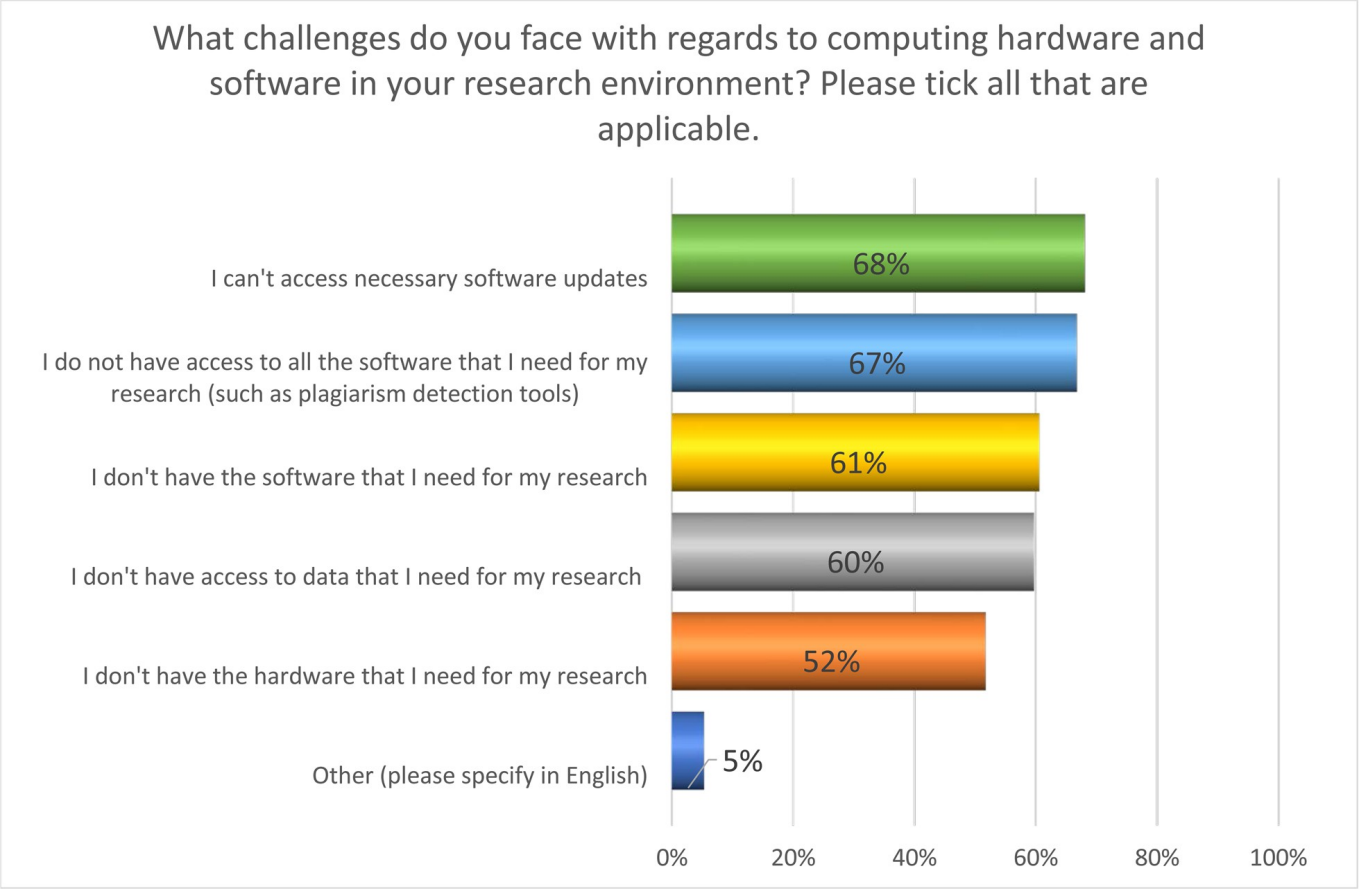
What computing hardware and software challenges are currently experienced by Sudanese academics?

The challenges of acquiring laboratory equipment and reagents is readily understandable given the import bans imposed by the sanctions regimes. In contrast, however, the challenges experienced in accessing information and communication technology (ICT) software are perhaps more surprising. In response to the question: what challenges do you face with regards to computing hardware and software in your research environment, the responses suggested widespread challenges (Fig 7).

**Fig 8:**
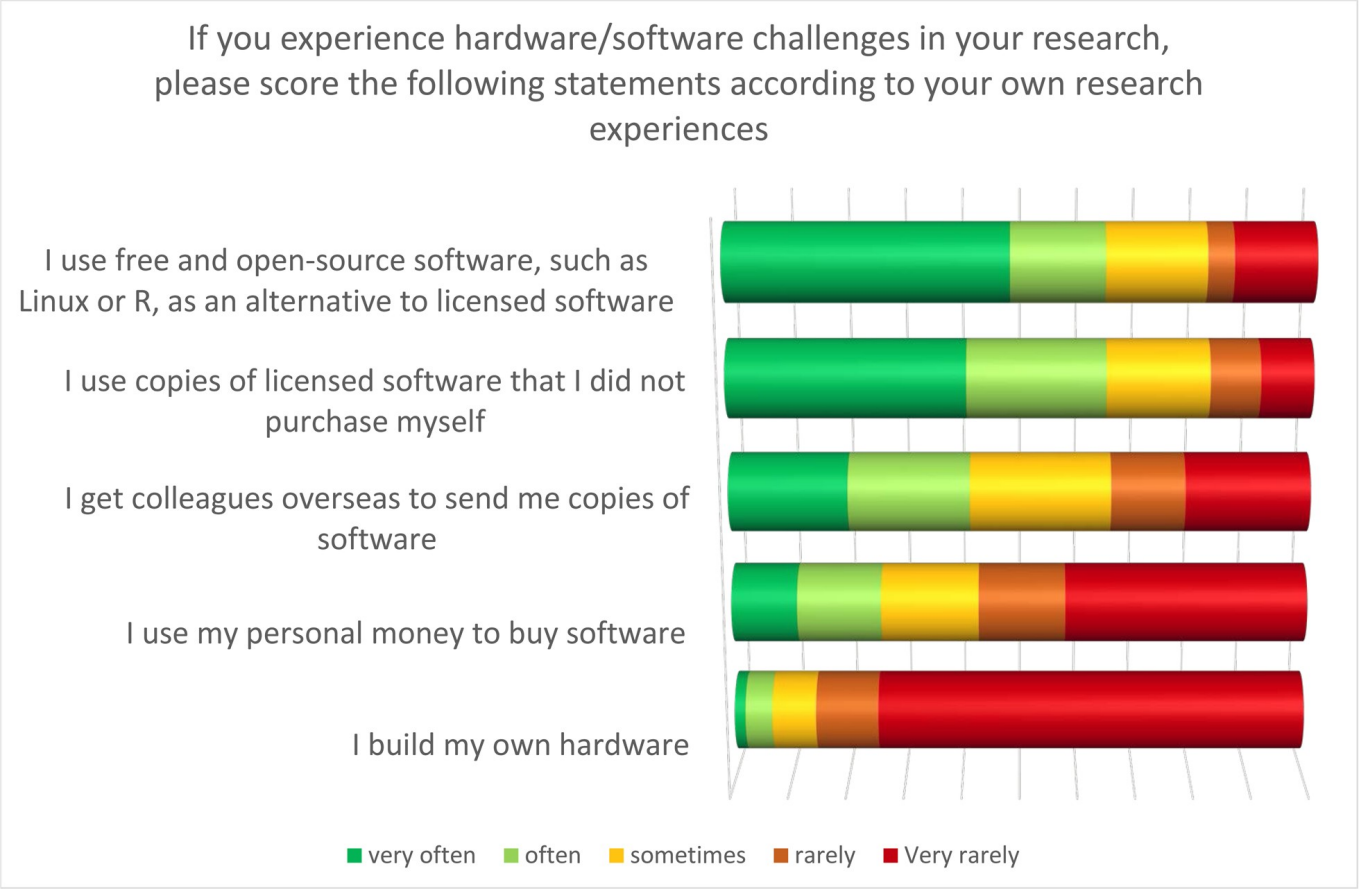
How do Sudanese academics currently overcome software challenges?

Interestingly, however, the two highest ranking statements were lack of access to software and lack of access to software updates (Fig 8). As Sudanese academics are currently unable to access products such as Kaggle due to the location of the owner company (Google), this is perhaps unsurprising. In the following question respondents admitted using software from a variety of sources, including free and open source software (FOSS) (64% agree/strongly agree), copies of proprietary software that they did not purchase (65% agree/strongly agree), or software sent from colleagues overseas (42% agree/strongly agree).

Based on these results it would appear that a significant number of participants were using pirated or unlicensed proprietary software. To a greater extent, this would therefore explain their reported inability to get software updates. Nonetheless, such results seem incompatible with the 64% of respondents claiming to use FOSS. It is, of course, possible that the term FOSS was not familiar to respondents, and that they took it to mean “free” rather than “open source” software (13). In order to probe this further, one of the authors based in Sudan (OK) attempted to download a commonly-used FOSS, R Studio. Without using a VPN she was unable to do so. Such experiences suggest that the access to FOSS is extremely problematic within Sudan.

#### Availability of data and papers under sanctions

As many publishers are commercial entities, they are subject to the same expectations of compliance as any other company. The vast majority of academic publishers are located in Europe and the US, causing the dissemination of research outputs from countries under sanction to also be problematic. This—particularly for those requiring an Article Processing Charge (APC)—can make them unwilling or unable to accept submissions from academics based in countries under sanction.

Probing further into the difficulties experienced by Sudanese researchers in accessing data online, respondents were asked to select statements that reflected their own personal experiences. 85.9% said they “struggled to pay membership fees to access data sites”. These difficulties came not only from a lack of research funds to cover such costs, but also due to the difficulty of making online financial transactions using Sudanese credit cards. Under sanctions, companies based in countries imposing sanctions on Sudan were not allowed to accept transactions from Sudanese accounts (Executive order 13067 [27]). It is important to recognize that this ban not only affected commercial institutions, but also include any academic network, databases or organization that banks or operates within a country imposing sanctions.

Another key challenge for Sudanese academics was IP blocking. 78.4% of respondents reported having “access blocked from certain sites because of their Sudanese IP address”. This practice has significant implications for access to online data, as seemingly open data resources because inaccessible due to the location of the researcher attempting to access them.

**Fig 9:**
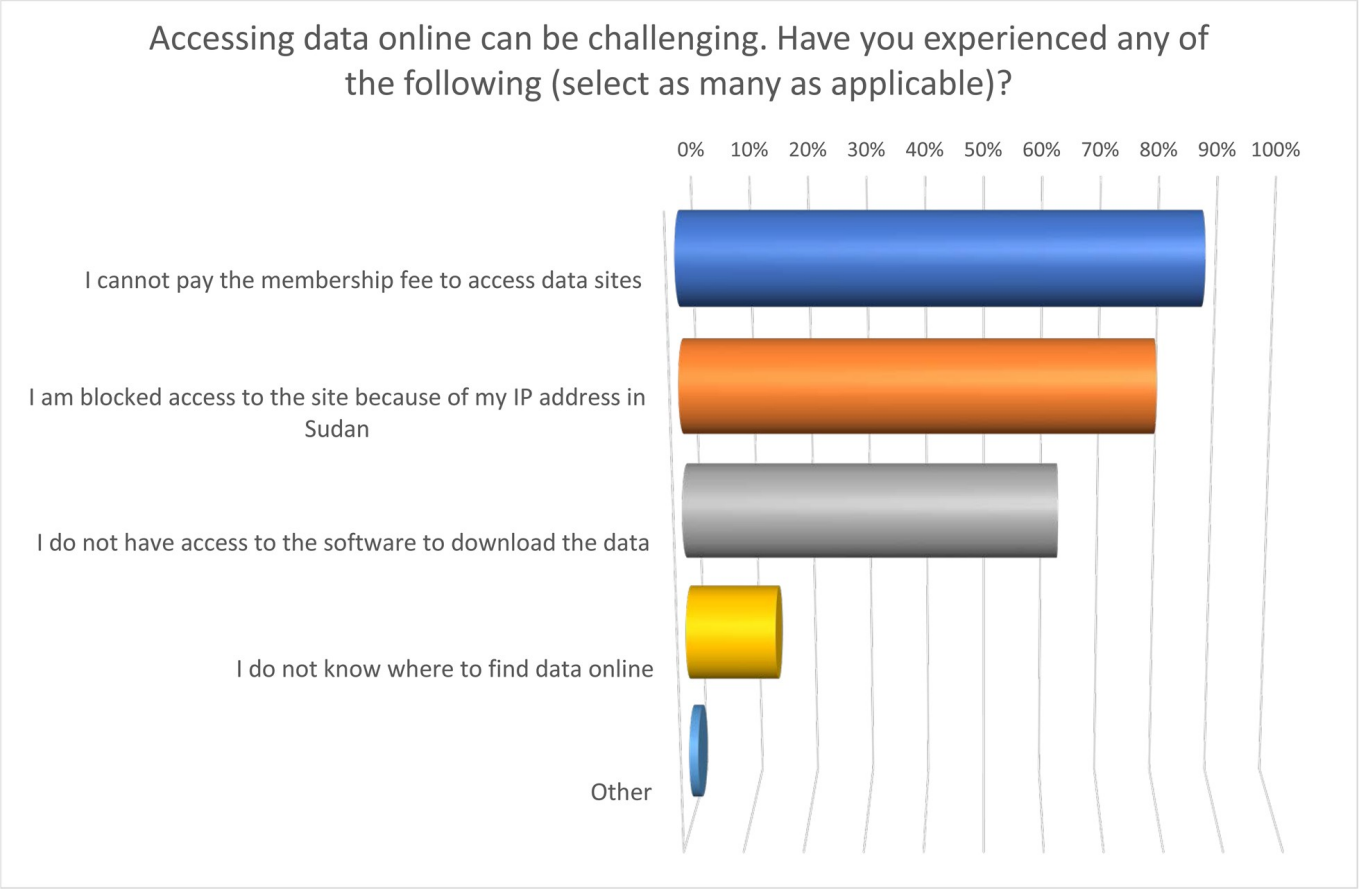
Problems of accessing publications and data experienced by Sudanese academics.

In addition, 62.31% of respondents reported not having “access to the software necessary to download the data” (Fig 9). Similar to the issues relating to membership fees described above, many Sudanese researchers struggle to purchase research software due to the difficulties of making international/online purchases. This can lead to situations in which they are unable to analyse the data they are able to gather due to the lack of appropriate software.

#### Risk averseness and collaborations under sanctions

It is important to recognize that many sanctions scenarios make specific exemption for certain areas deemed vital to the population, such as healthcare. Since 2013, the areas of exemption in the Sudanese sanctions have also explicitly included academic activities [28]. Nonetheless, changing sanctions regimes are not necessarily communicated to individual businesses—or indeed academic institutions. This can lead to lags in demonstrable changes [26]. Within our own collaboration, we experienced considerable difficulty making financial transactions between our universities in the UK and Sudan.

Perhaps even more problematic is that boundaries between research/commercial ventures, public/private/state activities, and education/innovation (in terms of activities, equipment utilized and research outputs) are increasingly blurred. This can mean that foreign businesses, collaborators and universities are unwilling to take the risk of possibly violating sanctions and preferentially avoid interacting with academics from countries under sanction. This “voluntary isolationism” extends beyond sharing/selling equipment, reagents, samples and hard/software and includes endorsing academic travel, collaboration, international networks and the acquisition of international research grants [12], [29].

**Fig 10:**
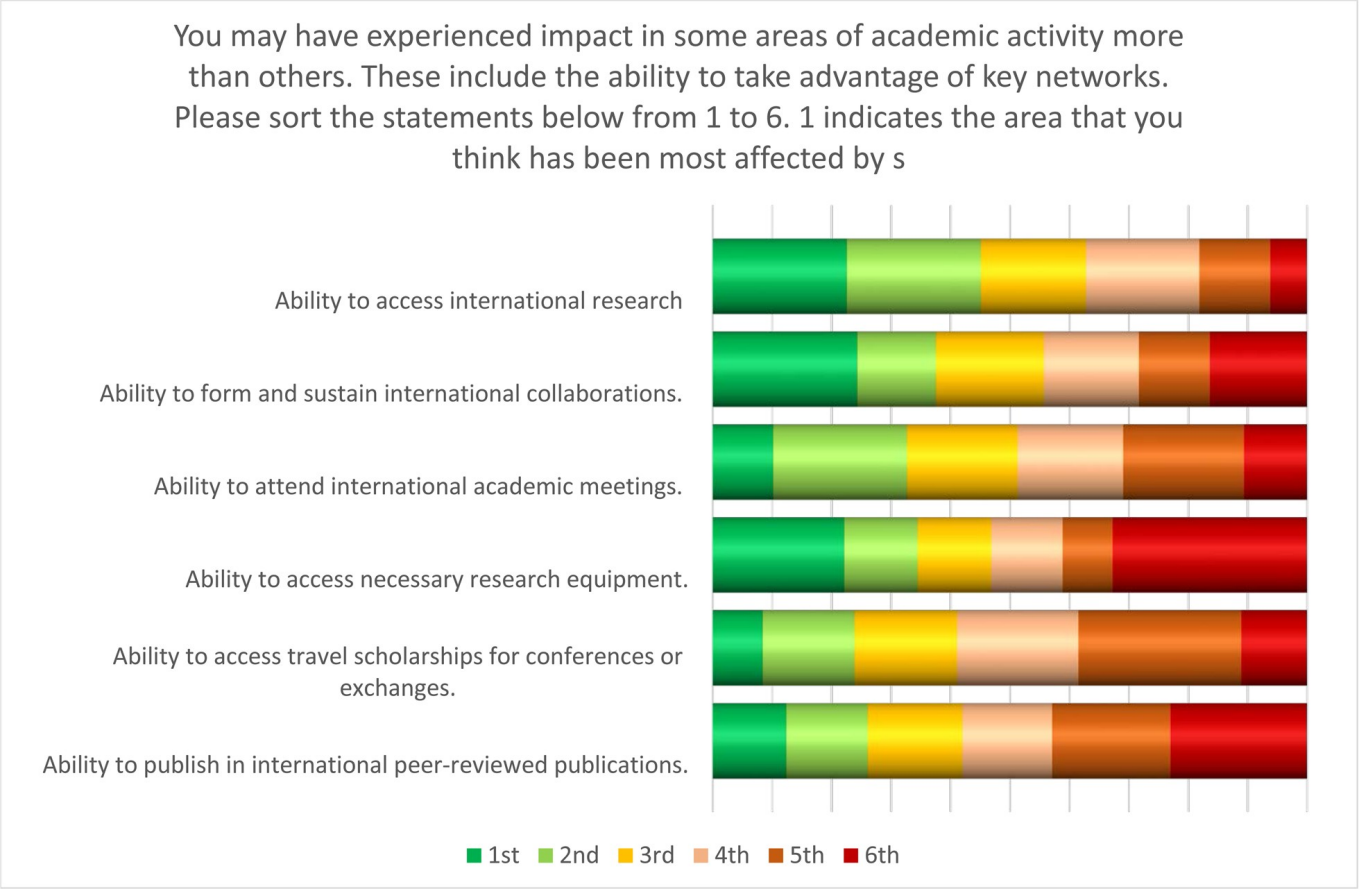
Difficulties experienced by Sudanese academics in engaging with the international research community.

No doubt in part due to lack of awareness and risk-averseness, many Sudanese academics reported having problems accessing international research funding, networks and collaboration. Respondents were asked to order statements about how they perceived sanctions to impact on their ability to interact with the global scientific community (Fig 10). The statement receiving the highest score was “sanctions affect my ability to access international research funds”. This observation is reinforced by the anecdotal evidence and weblinks offered by a number of respondents where funders explicitly stated that researchers from Sudan were ineligible to apply. These responses correspond to a later question about international funding, where only 12% of respondents said that they had received international funding. The second highest scoring statement was “sanctions affect my ability to form and sustain international collaborations”.

### Areas requiring further investment in order to facilitate the effective growth of Sudanese academia in the post-sanction era

The previous section outlines some of the (many) areas in which sanctions have impacted on Sudanese academia. The survey data strongly suggests that sanctions have had a marked, negative effect on the ability of Sudanese academia to develop along a similar trajectory as comparable, un-sanctioned countries. The data also suggests that Sudanese academia is not necessarily in the position to immediately take advantage of the recent changes in sanctions.

#### Relying on the resilience of systems is not sufficient

In the 20 years of economic sanctions, the Sudanese government has made efforts to sustain their academic community through targeted funding. This was evident in the responses to a range of questions. For instance, 50% of respondents agreed that they had “received some kind of fund/grant for your study or research during the period between 1993 and 2017”. Of these respondents, 73% of respondents agreed that they had the “opportunity to study for a postgraduate degree outside of Sudan”, and 40% said that they had received “national funding for the studies abroad”.

Similarly, as discussed above, 40.7% of respondents confirmed that they had received funding for research projects. Of those that had received funding between 1993 and 2017, 47.8% had received small national grants of under $10000. Larger grants were relatively scarce, with only 10.9% receiving national funding over $10000 and 17.4% international grants of the similar sizes. 58% of respondents said that they had “published a paper in a peer-reviewed journal”, but less than half of respondents had published more than 5 papers in the last 5 years.

These low levels of research outputs are also reflected in national data on publications. Scopus data suggests that following the start of sanctions in 1997, there was a visible dip in publications, including international co-authorships. The number of publications has risen tenfold since, most notably in the area of medicine, but in line with global averages since the explosion of the internet era. It is currently too soon to consider publication data since the end of sanctions in 2017—however, some preliminary data from Scopus suggests a significant rise in publications in computer science, engineering and mathematics. (Scimago Journal and Country Rank: Sudan, 2019, https://www.scimagojr.com/countrysearch.php?country=sd, accessed 26th July 2019)

In comparision, neighbouring countries such as Uganda and Ethiopia have experienced significantly more growth during the same time period. This suggests that sanctions have left a lasting mark on Sudanese academia, requiring Sudanese scientists to now play ‘catch up’ to their neighbours [29]. While the efforts of the Sudanese government to sustain the academic community are important, it is inappropriate to assume that these efforts have been sufficient to enable the national academic community to seamlessly re-integrate with global academia.

#### Major investments are needed

In response to an open question on the current issues relating to sharing and accessing research data online, one respondent said: *Most of the Sudanese community (including myself) is so behind on data usage and technology that even if it were open we wouldn't know how to use it efficiently*. This comment was representative of the 42 responses to the open question, suggesting a pervasive negativity regarding the current state of Sudanese academia. The idea that one “does not know what one does not know” encapsulates the feeling of being “left behind” by the international research community. The comments suggested that considerable investment in academic infrastructures were perceived as urgent.

In recent years there has been a rise in investments in African academia. Many funding bodies, such as the Wellcome Trust and the Gates Foundation, have earmarked considerable funds for funding African research projects. As the sanction regimes against Sudan dissipate, the eligibility of Sudanese academics for these funds can be expected. While this, of course, represents an important opportunity for research capacity building in Sudan, it must be recognized that such funding structures will not necessarily address the massive research technology deficit in the country. The majority of these funding initiatives offer project-specific funds that only allow project-related equipment purchases. They do not fund “core” laboratory equipment or other non-essential items [30]. While there are a small number of equipment brokerage and/or donation schemes, such as Seeding Labs and TReND in Africa, they are not large enough to be able to counter country-wide deficits immediately. If Sudan is to rely solely on these funding models, it is unlikely that infrastructural deficit will be quickly or efficiently addressed.

An additional complication of the sustained technological deficit relates to the “not knowing what one does not know” referred to above. For 20 years Sudanese academics have been marginalized from technological developments in their fields of research. This has left many without experience in the use of many of the research technologies currently in use. Effectively addressing the technological deficit thus also requires training for researchers in the use of the equipment they acquire. Moreover, there is a critical need for training for the support staff and technicians that will be responsible for the maintenance, calibration and repair of the research equipment.

#### Lack of contacts, networks and social capital

In a final open question, respondents were invited to identify “any other key issues challenging Sudanese academia”. Amongst other things, responses included language barriers/training in English communication (5 responses), learning how to collaborate (6 responses) and network at conferences (3 responses) as skills needed by Sudanese academics. These responses point to the perceived need for training in the soft skills that play an increasingly important role in modern academia. In particular, the responses highlighted the skills associated with direct communication with the international academic community.

The difficulties of international travel have left a lasting legacy for Sudanese academia. In the past 20 years, academics have struggled to attend international conferences and workshops. In addition to losing the opportunity to present their work and gain feedback on their research, academics have lost the chance to network amongst their peers. Such networking leads to greater research visibility, and often results in international collaborations.

Sudanese academics are thus placed in a difficult position. Sustained travel-related isolation means that many have few international contacts and active research networks. Moreover, lack of training in the “academic soft skills” can make them apprehensive of their future networking successes. Viewed collectively, it may be suggested that Sudanese academia currently experiences a deficit in “social capital”. As defined by Pierre Bourdieu (1983), this refers to "the aggregate of the actual or potential resources which are linked to possession of a durable network of more or less institutionalized relationships of mutual acquaintance and recognition” [31]. It is clear that efforts are needed to address this deficit in social capital through skill enhancement and increased travel, although clear strategies on how this could be done remain problematic. Indeed, increasing social capital cannot be reduced to increased opportunities to network, but the mutual adoption of a shared identity and understanding, norms and values, as well as shared trust, cooperation and reciprocity.

#### Skill shortages

In a number of questions, the survey respondents identified key skill shortages that they felt would impact Sudanese academia’s ability to benefit from the changing sanction regimes. Respondents were asked to rank statements in response to the question: “as sanctions are lifting, Sudanese academics will have increasing opportunities to engage with the international academic community. Do you feel that there are skill/resource gaps that you would like assistance with?”

**Fig 11:**
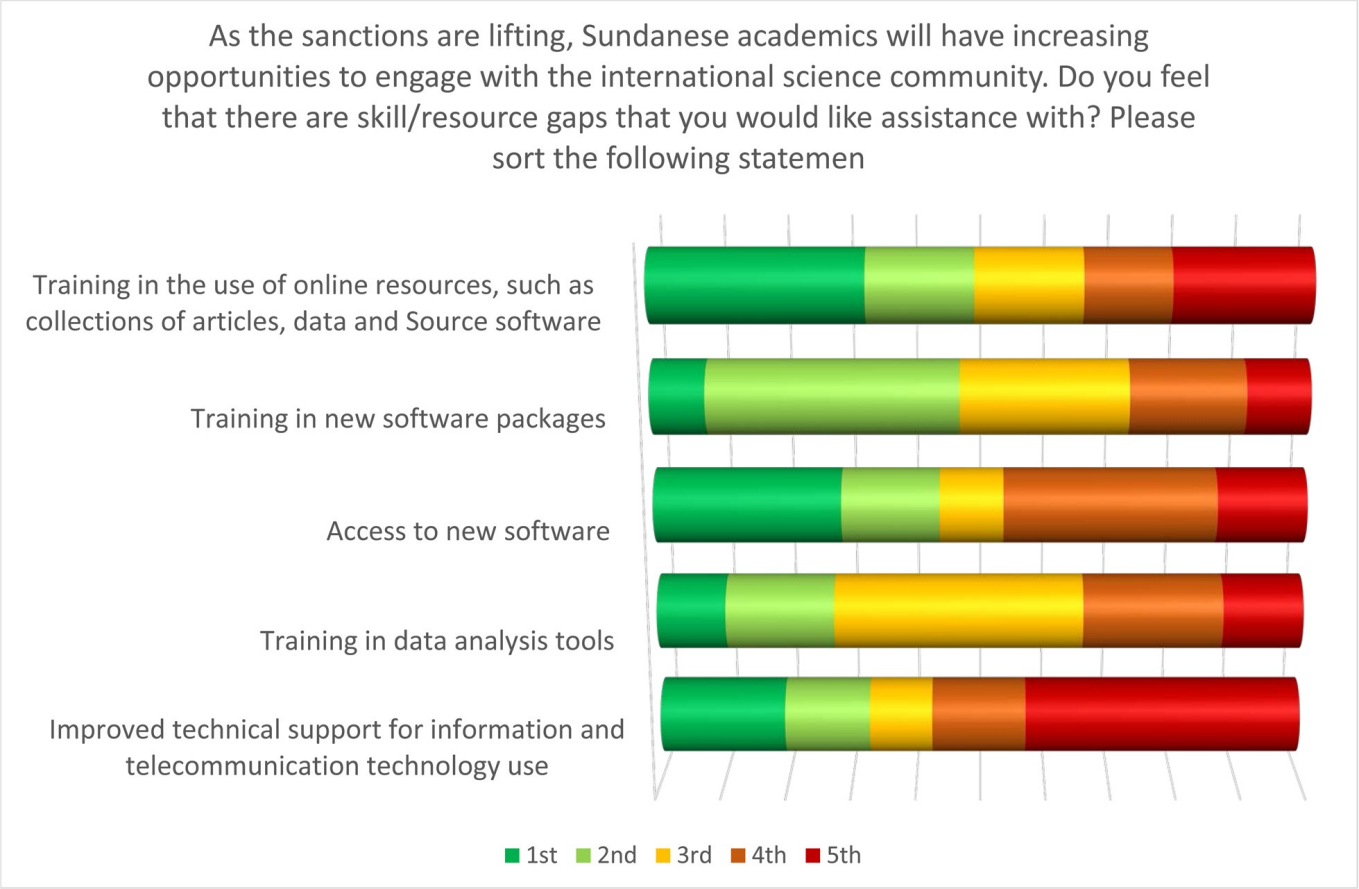
What skills are most necessary for Sudanese academics?

All the statements offered (Fig 11) received very similar weightings, indicating that respondents perceived the need for a range of interventions. Nonetheless, of all the statements the highest ranked was the need for “training in the use of online resources, such as collections of articles, data and FOSS”.

Similarly, when asked about communicating research to the global scientific community, the respondents selected “assistance in learning publishing/peer review processes” as the most important. Similarly, a question on research data had the need for “training in data management skills” as the most critical requirement. Together these responses suggest that the sustained isolation of the Sudanese academic community has left skill gaps in the ability to not only communicate research findings, but to make use of the research findings of others.

The survey concluded with an open question where respondents were invited to add any other issues they viewed as currently challenging Sudanese academics. Respondents (73 respondents) identified language barriers/training in English communication (5 responses), as well as training in “scientific thinking” (4 responses), writing papers (10 responses), and ethics training (4 responses) as of importance. Respondents also identified the need to learn how to collaborate (6 responses) and to network at conferences (3 responses) as skills needed by Sudanese academics. From the responses it is evident that the respondents considered a range of the so-called “soft skills” as vital for academic careers. In particular, skills associated with direct communication were deemed as lacking, undoubtedly linked to the impact that travel bans have had on international conference attendance and collaborations.

## Discussion

### A rapidly changing milieu

In January 2017, the US began to lift economic and trade sanctions on Sudan, due to cooperation from the Sudanese government in fighting terrorism, reducing conflict, and denying safe haven to South Sudanese rebels, as well as improving humanitarian access to people in need. In October 2017 the US permanently lifted all 1997 sanctions after Sudan cut all ties with the North Korean regime of Kim Jong Un [32]. While other countries maintain weapons embargoes against Sudan, many have similarly adjusted their economic sanctions. The sanctions imposed by the United Nations Security Council in relation to the Darfur conflict remain in place [33].

While the lifting of US sanctions was met with optimism, the hoped-for economic regeneration remained absent. Indeed, since 2017 the Sudanese economy has been plagued by sluggish growth and hyper-inflation. Moreover, Sudanese citizens continued to report continued difficulties accessing foreign goods and services. According to a recent news report, “Sudanese officials blame the US—suggesting that Washington has not properly spread the word that there is no longer any risk involved in doing business in Sudan. Others, though, feel that the Sudanese government used the sanctions to mask its own responsibility for the deteriorating living standards” [33].

In December 2018 a series of demonstrations broke out in a number of Sudanese cities, due at least in part due to the rising cost of living and the national economic [34]. These protests rapidly turned into a nation-wide movement demanding the resignation of President Omar al-Bashir. In April the military removed al-Bashir from power, but failed to implement a civilian-led transitional government [35]. Following almost 7 months of extreme unrest a deal was agreed between the military and civilian protesters and a period of transition was entered leading to elections in 2022 [36].

### Going forward

Sudanese academia seems at an impasse. 20 years of sanctions have had a marked effect on the academic community, research infrastructures and practices, but the recent lifting of these sanctions will not return the situation of “business as usual”. This survey demonstrated that the imposition of sanctions will have long-term effects far into the sanction-free future. The sanctions have isolated, and will continue to isolate, Sudanese researchers.

While Sudan is gradually being included on funding schemes (such as the UK Global Challenges Research Fund), there is a pervasive silence about whether national governments (and the research communities in these countries) owe Sudanese academia some kind of reparative investment. Our survey clearly shows that assuming the Sudanese academic community will seamlessly be able to re-integrate and be instantly globally competitive are both naive and lazy.

The first set of results presented data about the long-term effect of sanctions on academic growth. The second set of results flagged areas urgently needing attention, such as investments in technological infrastructures, up-skilling activities and networking opportunities. While these suggestions are very similar to other capacity building activities on the African continent, they differ in terms of chronology and moral justification. The former highlights the urgent need for these interventions to ensure that the negative long-term impacts of sanctions are minimized as soon as possible. Ensuring a timeous response will enable academic systems to recover effectively from the enforced isolation.

The latter difference refers to the reason why such activities should be undertaken. Capacity building activities amongst LMIC academia are normally presented according to the more general justifications for aid [37], whereby both contributors and recipients derive benefit from the interaction through good-will, mutual collaborations and a broader community of expertise. In addition to these humanitarian and distributive justice arguments, the responsibility for post-sanction activities must be recognized to carry another form of moral duty as sanctions are understood as a form of aggression.

### Jus post poenas

In modern warfare the concept of *jus post bellum* refers to the post-battle activities aimed at building a “just and lasting peace” [38]. Scholars such as Brian Orend have proposed a deontological underpinning for a three-pronged approach to the morality of armed conflict [39]. Appropriate post-war actions include a range of activities that focus on stabilising and rebuilding war-torn societies and restoring/implementing democratic governance [38], [40], [41]. These activities are not only recognized as vital for sustaining the peace within the affected country, but an important aspect of the victor’s just war defence for military intervention.

Despite economic sanctions being viewed as the liberal alternative to war [1], there has been little systematic discussion on what would constitute peace-building activities for sustained economic sanctions would constitute. To paraphrase the terminology above, what would be a *jus post poenas* (we use the Latin term *poena* to refer to sanctions, which demotes a punishment, penalty, or “to pay the price”). As a result, there is little systematic evidence detailing the types of support offered to nations recently emerging from sanctions. Nonetheless, it is important to recognize that the lifting of sanctions does not guarantee that countries automatically return to “business as usual”. Particularly in the case of long-term, widespread sanctions there could be extremely significant and long-lived implications. In addition to the obvious impact on the national economy, these could include:

- Social systems needing to realign with global trends
- Outmoded systems of technology
- Lack of international trade/collaboration

This lack of post-sanctions peacebuilding activities is exacerbated by the highly variable response from the international business community. The lifting of economic sanctions is unevenly publicized, and the implementation largely up to the individual company. Many businesses will thus delay updating sanction information—either from lack of knowledge, apathy or excessive caution. As a result, the impact of sanctions can be perpetuated beyond their legitimate end as companies neglect to update their financial practices [42].

All of these issues have significant implications for the academic community. Indeed, lifting sanctions cannot be seen as automatic reintegration into the international science community. Depending on the length and severity of a regime of sanctions, academic systems could face long-term physical, social and regulatory challenges. Physical challenges could include outdated research infrastructures that require considerable financial investment to modernize. Social challenges could include a dearth of international collaboration, lack of international recognition for existing research, lack of engagement with current research trends, and a scarcity of key skills (such as data science) that are defining modern research. Regulatory complications could arise from a lack of alignment between in-country regulations governing research (such as biosafety and biosecurity) and international standards, which could hamper future funding and collaboration relationships. Moreover, as mentioned above, widespread tardiness within commercial companies to remove sanction-related restrictions in their financial systems could mean that academics continue to face challenges in making international/online purchases for reagents and equipment.

Recognizing these multifaceted challenges raises important questions. Most broadly, it necessitates that we question what *jus post poenas* should mean for situations of sanctions—both for those imposing the sanctions, and for those receiving them. In such cases, if education, research and innovation are recognized as important elements of economic growth, is there an expectation that *jus post poenas* activities extend to supporting the reintegration of previously-sanctioned academics into the international academic community. If so, does the responsibility lie solely at a national level, or does the international academic community bear some responsibility towards supporting academic re-integration? This section briefly considers four questions relating to developing a *jus post poenas* for academia recovering from sanction regimes. The relative silence about how to re-integrate the Sudanese academic community—and who is responsible–must be seen as symptomatic of a broader problem with economic sanctions, namely the lack of *jus post poenas* in addressing the aftermath of sanction regimes.

### Responsibility: Finding where the “buck stops”

One of the most obvious problems of assigning a *jus post poenas* responsibility for sanctions is the nebulous nature of both the implementer and the recipient. In large part, this is due to the pervasive and non-specific effect of economic sanctions. Indeed, as we highlight in this paper, the academic community was not the explicit target of the sanctions imposed on Sudan (and, indeed, since 2013 were explicitly exempted from these sanctions). Petitioning implementer nations for financial investments in academic capacity building are likely to be fraught with attempts to assign, or shift, blame.

Another key problem relating to the non-specific impact of economic sanctions involves the inevitable “prioritisation” of development targets that would guide any *jus post poenas* activities. If any money is dedicated, it is likely that this will be directed to peace-building activities, reinvigorating manufacturing or developing structures to support democracy. Such prioritization reflects the close relationship between the security and peace building communities [43], from which academia is largely absent. This is not to say, however, that this exclusion is inevitable. The prominent role that RDI play in national development strategies [44] suggest that future conversations are possible … but they do not occur yet.

Who, then, bears the responsibility for addressing both the immediate and long-term impact? If, as suggested, it is unlikely that there will be considerable traction from national governments, where should pressure be applied? We suggest that the responsibility lies with the global academic community—researchers, funders and related stakeholders. The modern milieu of research is characterised by the Open Science movement that priorises the free and open sharing of scientific resources in a manner uninhibited by borders, disciplines or societal positions [45]. Open Science is underpinned—and dependent on—by a commitment to egalitarianism. Highlighting that sanctions thwart this egalitarian ideal should place the responsibility for *jus post bellum* firmly at the feet of the global science community, and places a moral duty not to ignore the insidious barriers that sanctions erect in the seemingly open world.

### Activism: Addressing the “transformation lag”

One of the difficulties of addressing the long-term impacts of economic sanctions is the uneven way in which information about the lifting of sanctions is disseminated to commercial (and non-commercial) entities. Indeed, Sudanese officials suggest that the US has not properly spread the word that there is no longer any risk involved in doing business in Sudan [42]. Anecdotal evidence from Sudanese academics suggest that many of the companies they interact with maintain the trade restrictions they utilized during the sanction period. A key element of effective *jus post poenas* would thus involve ensuring that companies providing academic resources are cognizant of sanction changes.

For countries such as Sudan, motivating companies to update, change or refresh their current business strategies in their favour is complicated by the relative size of the Sudanese academic market. As it continues to represent a very small portion of the global research market, there is little incentive for these companies to invest time and financial resources in overhauling their business practices. Nonetheless, the ideology of the global (Open) academic community recognizes that academic communities around the world deserve the same opportunities to flourish as academics, regardless of context.

### Empathy: Committing beyond sanctions

In many cases, sanctions are symptomatic of broader national challenges. Indeed, the removal of sanctions does not automatically guarantee that researchers will be working in stable and flourishing national contexts. In many cases the imposition of sanctions is not solely about ideological differences, but symptomatic of activities resulting from poor governance, lawlessness and state mismanagement.

**Fig 12:**
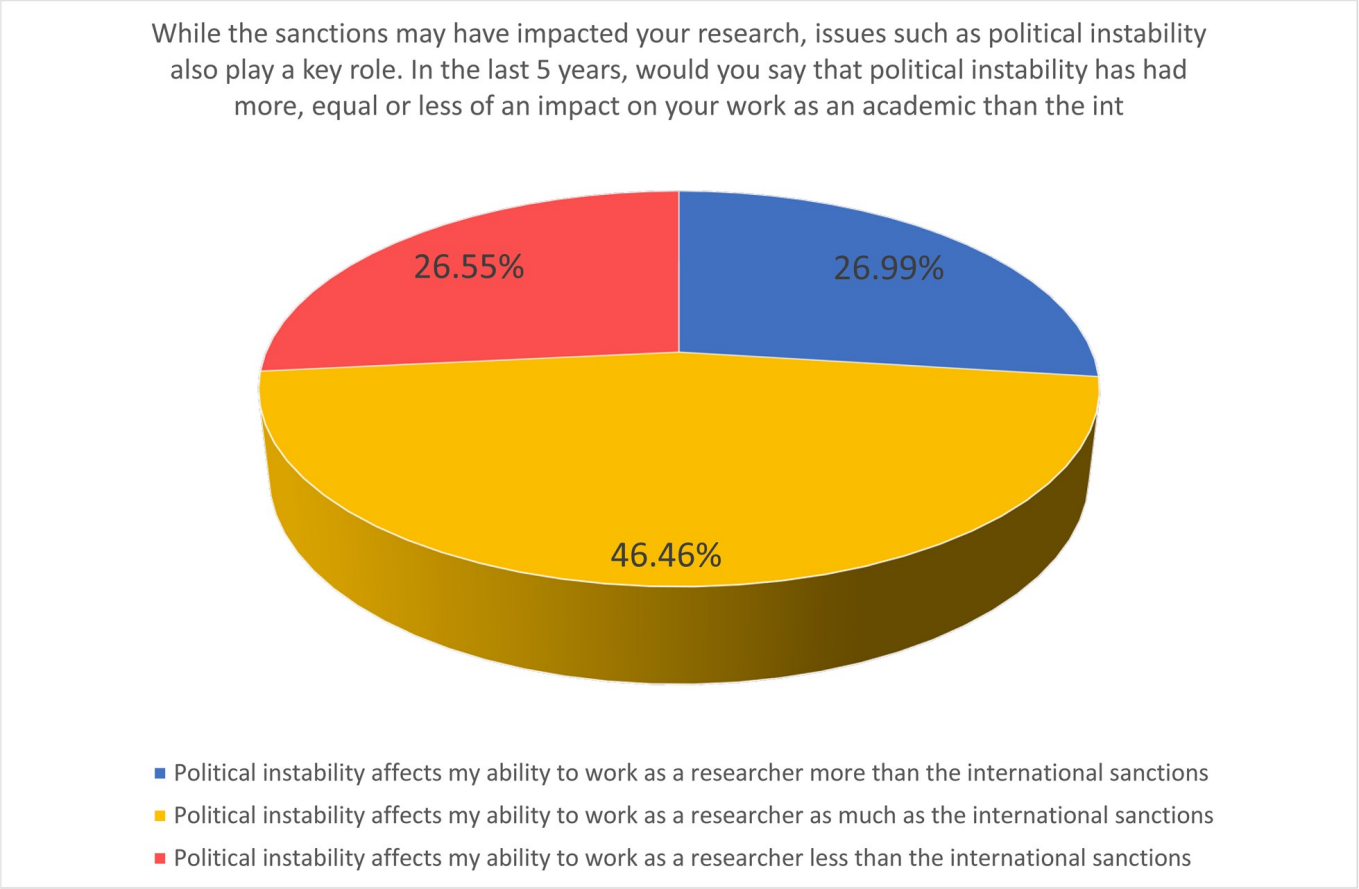
Political instability is as impactful as sanctions on Sudanese academia.

Such is undoubtedly the case in Sudan, a nation that has recently experienced extreme political turmoil. In situations such as this, it is unlikely that the academic system will receive the necessary support needed for rejuvenation in the near future. This observation is supported by survey responses, where respondents ranked political instability as equally disruptive to their work as academics (Fig 12). It is highly likely that they will continue to face considerable challenges in the post-sanction future. Such challenges, unfortunately, do not necessarily go away overnight.

Further compounding the post-sanction challenges of Sudanese academics is the continued hyper-inflation and economic instability [46]. The currency has gone through a period of rapid devaluing and Sudanese academics—regardless of their new ability to apply for international grants—will struggle with problems of budgeting and international purchasing (due to lack of forex). Such situations significantly complicate efforts to build research capacity. This financial uncertainty undoubtedly contributes to the persistent “brain drain” experienced by Sudan, that is similarly undermines other fragile academic systems. The 2016 UNESCO Science Report: Towards 2030 drew attention to the impact of conflict and brain drain on development. It pointed out that between 2002 and 2014, Sudan lost more than 3,000 junior and senior researchers to migration.

Asking the global academic community to engage in *jus post poenas* activities—in Sudan and in other countries—is thus not likely to be straightforward. The challenges associated with conducting research/networking/educational activities in unstable states are well recognized. In order to ensure interventions that are effective and have longevity thus requires a firm moral commitment, practical support and pragmatic experience sharing. Effective *jus post poenas* needs to be a long-term strategy, and cannot be thought of as once-off activities or a brief financial commitment.

## Concluding comments

We recognize that sanctions are a difficult topic to address. They sit at the nexus between security, national identity and social values and raise uncomfortable tensions between individual and academic identities. These tensions should not be minimized, and the individual interpretations of what constitutes “right action” should be respected. Nonetheless, the immediate and long-term impact of economic sanctions have the potential to damage present and future academic research in affected countries. In turn, this has widespread implications for economic and social development. We therefore advocate strongly for discussions about how *jus post poenas* activities could be developed to ensure that the impact on academia is minimized.
