# Correction: Economic sanctions and academia: Overlooked impact and long-term consequences

**DOI:** 10.1371/journal.pone.0225277

**Published:** 2019-11-18

**Authors:** Louise Bezuidenhout, Ola Karrar, Javier Lezaun, Andy Nobes

The following information is missing from the Funding statement: A Quality Related Global Challenges Research Fund award was made to JL for the project “International Sanctions and Global Science,” from a block grant made to the University of Oxford by Research England.
